# Multi-degrees-of-freedom soft robotic ankle-foot orthosis for gait assistance and variable ankle support

**DOI:** 10.1017/wtc.2022.14

**Published:** 2022-08-01

**Authors:** Carly M. Thalman, Tiffany Hertzell, Marielle Debeurre, Hyunglae Lee

**Affiliations:** Neuromuscular Control and Human Robotics Laboratory, Ira A. Fulton Schools or Engineering, Arizona State University, Tempe, Arizona, USA

**Keywords:** soft robotics, assistive robotics, rehabilitation robotics, gait assistance, gait rehabilitation, ankle robot

## Abstract

This paper presents the design, modeling, analysis, fabrication, and experimental characterization of the soft robotic ankle-foot orthosis (SR-AFO), which is a wearable soft robot designed for ankle assistance, and a pilot human study of its use. Using two novel pneumatically powered soft actuators, the SR-AFO is designed to assist the ankle in multiple degrees-of-freedom during standing and walking tasks. The flat fabric pneumatic artificial muscle (ff-PAM) contracts upon pressurization and assists ankle plantarflexion in the sagittal plane. The multi-material actuator for variable stiffness (MAVS) aids in supporting ankle inversion/eversion in the frontal plane. Analytical models of the ff-PAM and MAVS were created to understand how the changing of the design parameters affects tensile force generation and stiffness support, respectively. The models were validated by both finite element analysis and experimental characterization using a universal testing machine. A set of human experiments was performed with able-bodied participants to evaluate: (a) lateral ankle support during quiet standing, (b) lateral ankle support during walking over compliant surfaces, and (c) plantarflexion assistance during push-off in treadmill walking. Group results revealed increased lateral ankle stiffness during quiet standing with the MAVS active, reduced lateral ankle deflection while walking over compliant surfaces with the MAVS active, and reduced muscle effort in ankle platarflexors during 40–60% of the gait cycle with the dual ff-PAM active. The SR-AFO shows promising results in providing lateral ankle support and plantarflexion assistance with able-bodied participants, which suggests a potential to help restore the gait of impaired users in future trials.

## 1. Introduction

Human locomotion is one of the most critical physical tasks for an individual to maintain independence and achieve the desired activities of daily living (Spector and Fleishman, 1998). The human ankle joint is a critical point of rotation and weight translations, and is one of the major contributing factors to assist in forward locomotion and postural stabilization (Mueller et al., 1995; Sawicki and Ferris, 2009). Two major contributors to successful forward locomotion are plantarflexion and medial/lateral ankle stability for balance throughout walking. Ankle plantarflexion is responsible for 45% of the power behind moving the body forward during walking (Winter, 1983; Farris and Sawicki, 2011). Push-off is the stage of human gait, which is a major contributor to forward propulsion during walking, and occurs at roughly between 45 and 60% of the gait cycle (Winter, 1983; Winter and Sienko, 1988).

There are many factors that can impact an individual’s ability to achieve natural and comfortable mobility. Among the most common factors are injuries from trips or falls, neuromuscular conditions, and neurological disorders, and many of those with a history of ankle injury (or injuries) face an increased risk of reoccurring injuries (Yeung et al., 1994). Chronic ankle instability is a common ankle impairment that affects the medial/lateral ankle stability and increases the risk of future ankle sprains and injuries (Garrick, 1977; Venesky et al., 2006). Ankle sprains usually occur when there is a sudden instance of inversion due to unanticipated lateral ankle buckling (Garrick, 1977; Venesky et al., 2006).

Ankle-foot orthoses (AFOs) are the most commonly used orthoses available to patients, accounting for as many as 26% of all orthoses provided to patients in the United States (Whiteside et al., 2007; Lusardi et al., 2013). For example, AFOs are commonly prescribed for recovering survivors with symptoms of hemiparesis, which affects around 80% of stroke survivors (Lusardi et al., 2013; Sankaranarayan et al., 2016; Cogollor et al., 2018). While there are many versions of AFOs available on the market, they are largely made out of rigid materials like plastics and carbon fiber (Whiteside et al., 2007; Lusardi et al., 2013).

With the rise in popularity of the wearable robotic industry, new robotic devices are being created to replace existing assistive and rehabilitative technologies (Park et al., 2014; Lee et al., 2016; Ren et al., 2017; Chung et al., 2018; Kwon et al., 2019; Shi et al., 2019). The field of assistive soft robotics has gained popularity within the wearable robotics community (Bao et al., 2018; Thalman and Artemiadis, 2020). Previous works have made significant advancements to substantiate the recent popularity of the use of soft, compliant materials to create wearable robots that are lightweight, comfortable, and effective in providing assistance to the user (Malcolm et al., 2017; Chung et al., 2018; Kwon et al., 2019; Thalman et al., 2019). Integrating textiles and fabrics into a wearable robot can help eliminate some of the drawbacks seen with rigid exoskeletons such as size, weight, and cost (Asbeck et al., 2013; Park et al., 2014; Malcolm et al., 2017). This is especially critical when assisting a joint such as the ankle, where gait dynamics can be heavily affected by even slight changes to external conditions (Browning et al., 2007).

**Figure 1.**
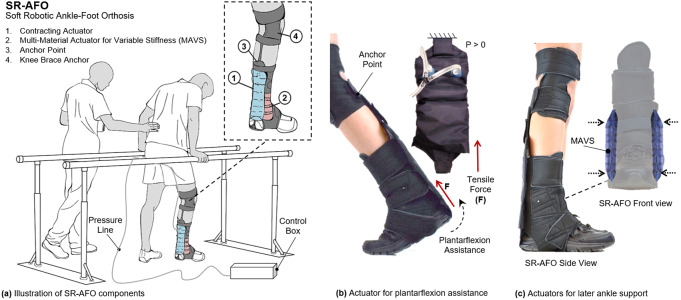
(a) The concept illustration of a soft robotic ankle-foot orthosis (SR-AFO) that assists walking with active ankle plantarflexion assistance as well as medial/lateral ankle support. Since the SR-AFO is designed to be used in its current stages as a rehabilitative device in clinical trials, the goal is to have the participant in a rehabilitative space already equipped with pressure lines, and the hardware needed to control pressure, timing, and record data from each session will sit beside the participant. Ideally, in most cases, there would be no compressor in this setup, rather the electropneumatic hardware box would connect directly to line pressure from the wall. (b) The actuators used for plantarflexion assistance are placed on the back of the leg, and contract to pull the heel upward. (c) The actuators to provide lateral ankle support are placed on either side of the ankle joint, and act as a brace when active to provide variable stiffness to the joint.

This paper presents the SR-AFO exosuit, which integrates soft, pneumatic actuators made of garment-like fabrics. The SR-AFO utilizes principles of soft robotics to help alleviate troublesome attributes of other traditional AFO solutions. It integrates two novel soft actuators and operates in multiple degrees of freedom of the ankle, providing both assistance in ankle plantarflexion in the sagittal plane and medial/lateral ankle support in the frontal plane (Figure 1). The SR-AFO is designed to provide active assistance at the precise intervals where it is needed without impacting the comfort or range of motion of the wearer. This paper presents the design, modeling, analysis, fabrication, and experimental characterization of the two soft actuators for the SR-AFO, and a pilot human study of its use.

The goal and motivation of designing the SR-AFO was to create a solution that would provide both the lightweight wearable orthoses such as the rigid ankle braces, with the dynamic support of a robotic ankle orthosis. The focus and novelty of this work is in the design of a device that could provide dynamic support and assistance to the user without adding excessive mass to the ankle during lower extremity tasks, with an easy don/doff process, which is unlike anything else currently used in ankle-targeted or gait rehabilitation.

## 2. Design, Modeling, Analysis, Characterization, and Fabrication of the SR-AFO

### 2.1. Actuator Concept and Design

**Figure 2.**
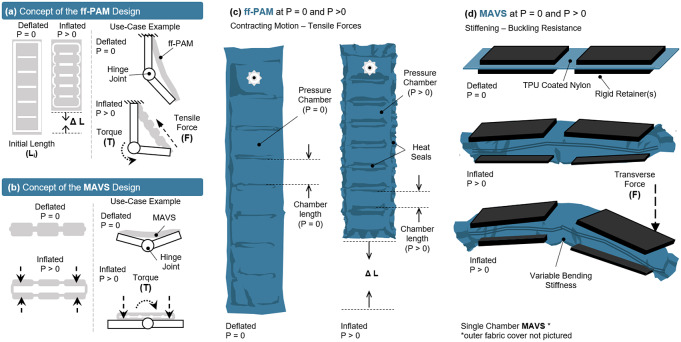
The concept illustration of (a) the flat fabric pneumatic artificial muscle (ff-PAM) actuator in a simplified geometry to show inflated and deflated states, as well as a basic diagram of how the actuator provides joint torque. (b) The multi-material actuator for variable stiffness (MAVS) is shown in a simplified form in the inflated and deflated states, as well as in a simple diagram showing how the MAVS can brace a joint against buckling. Deflated ( 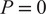 ) and inflated ( 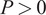 ) states of (c) the ff-PAM actuator and (d) the MAVS actuator are shown in more detail, with material layers, seams, and basic function.

The SR-AFO is a soft, wearable ankle robot that is made entirely of fabric materials such as Neoprene, Spandex, and Nylon. The combination of these materials forms a pattern that creates the main body of the SR-AFO, which can be worn over the user’s athletic shoe and can accommodate most adult shoe sizes. The SR-AFO comprises of two sets of soft actuators, that is, flat fabric pneumatic artificial muscle (ff-PAM) and multi-material actuator for variable stiffness (MAVS) actuators, which serve different primary functions in the overall performance of the soft exosuit. Upon pressurization, the ff-PAM contracts and increases tensile force (Figure 2a,c), whereas the MAVS increases the lateral stiffness (Figure 2b,d).

### 2.2. Analytical Modeling

The analytical models for the ff-PAM and MAVS actuators were created using the geometric programming of materials, which has been shown to be a useful approach for modeling textile-based soft pneumatic actuators (Niiyama et al., 2015; Thalman et al., 2019; Kwon et al., 2020). The assumptions used in modeling textile-based actuators include: (a) soft materials are inextensible, (b) soft materials assume common geometric shapes when inflated, and (c) fully inflated soft segments assume a “rigid” behavior at maximum pressure.

#### 2.2.1. Modeling of flat fabric PAM

**Figure 3.**
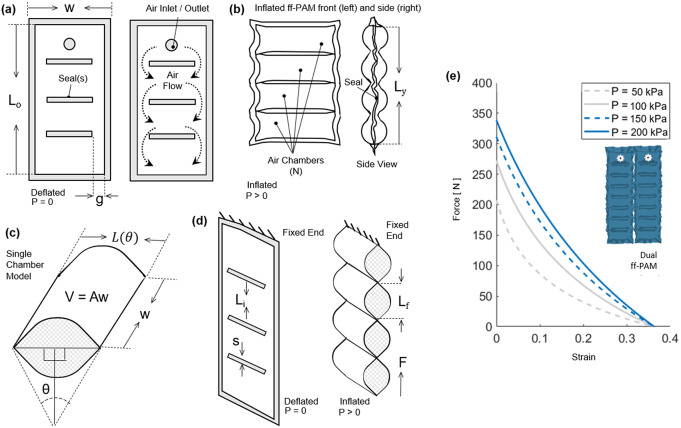
(a) The frontal view representation of the ff-PAM at 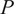 = 0, which indicates its geometries and the path of airflow within the chambers. (b) The frontal view of the ff-PAM at 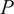 > 0 where the length and geometries are altered as a result of pressurization. (c) The cross section of a single chamber inspired by previous model iteration of inflatable pouches (Niiyama et al., 2015). (d) The isometric view of the ff-PAM in deflated and inflated states, where 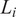 and 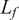 are the initial and final lengths of 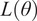 , respectively. (e) The theoretical tensile force versus strain curve for the dual ff-PAM actuator, with eight chambers, at pressure levels of 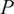 = 50, 100, 150, and 200 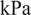 resulting from the analytical model. Increasing pressure level result in a more stable and linear response in actuator force profile.

The first actuator introduced was the ff-PAM. The model used to represent the ff-PAM is governed by the following set of equations (Figure 3; Niiyama et al., 2015): 
(1)

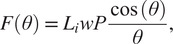



(2)

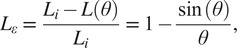

where 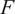 is the tensile force, 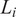 is the original length of each chamber, 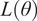 is the length of each chamber after inflation, 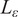 is the contraction ratio of the actuator, 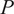 is the supply pressure, and 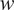 is the width of the actuator. Estimation of force output is based on the laws of conservation of energy. The relation between the supplied pressure and the resultant force output can be expressed as
(3)

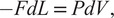

 where 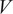 is the internal volume of the pneumatic chamber. Both 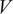 and 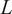 can be expressed as a function of 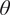 , and the expression can be written as
(4)

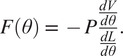

In order to determine the contraction in terms of strain ( 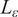 ), a corrected version of 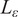 is used ( 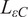 ):
(5)

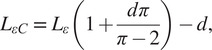

where 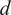 is a correction coefficient as described in (Niiyama et al., 2015) to account for some of the flexibility and compliance of the soft, thin textiles used to fabricate the actuator, which can affect the resulting strain. The length of the entire actuator, including all chambers of the actuator, with all chambers of the actuator was later optimized via finite element analysis (FEA) using the following conditions:
(6)

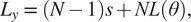

where 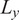 is the total length of the ff-PAM, 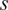 is the distance between each chamber created by the heat seal, and 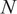 is the total number of chambers.

The number of chambers used in the ff-PAM was set to 8 ( 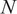 = 8), considering overall length, contraction performance, and robustness of the actuator. In addition, while the dual actuator configuration increased the total volume of the active ff-PAM setup compared to the single actuator, the increase in tensile force output was considered valuable for the application of plantarflexion and push-off assistance, and the SR-AFO exosuit used the dual ff-PAM configuration.

The force versus strain curves for the dual ff-PAM design with 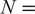 8 is obtained using the analytical model at four pressure levels (50, 100, 150, and 200 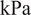 ; Figure 3e), which show that the tensile force of the ff-PAM increases with increasing 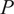 . The dual ff-PAM actuators were predicted to reach 307 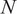 and 348.3 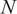 at the theoretical point 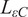 = 0 at 150 and 200 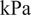 , respectively.

#### 2.2.2. Modeling of multi-material actuator for variable stiffness

The second actuator introduced in the SR-AFO was the MAVS, which integrated rigid components into a soft, fabric-based actuator. Inspiration for the integration of multi-segment, multi-material for the MAVS actuator was drawn from several previous works, such as the sliding layer laminate actuator design presented by Jiang and Gravish (Jiang and Gravish, 2018). This design focused on a three-layer laminate actuator that varies stiffness based on the ratio of the size of each exposed material when subjected to transverse load. The stiffest condition was achieved when there was a total misalignment of the layers, where the exposed soft material was minimized. Similarly, the smallest stiffness was achieved when the layers were stacked in vertical layers with no human error in fabrication misalignment, where the exposed soft material was maximized. The MAVS design was implemented with these physical characteristic behaviors in mind. The rigid retainers embedded in fabric were placed on the outside of the layer of inflatable actuator. The rigid pieces were aligned on the top and bottom of the actuator. A single segment of the MAVS weighed between 31.2 and 89.3 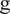 , depending on the configuration used.

**Figure 4.**
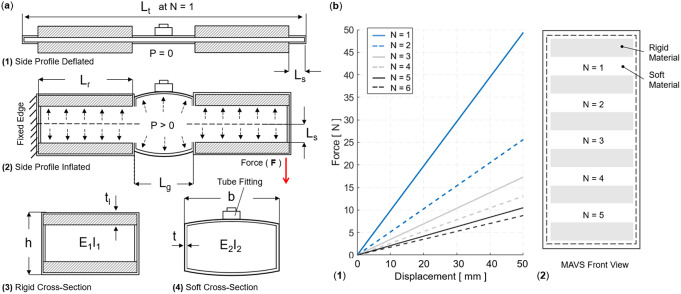
(a) The multi-material actuator for variable stiffness (MAVS) actuator when deflated and inflated. A cross section view of the MAVS actuator for the rigid and soft parts. 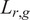 is the length of each segment, where 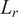 is representative of the length of the rigid sections, and 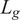 is the length of the soft segment. 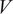 is the deflection of the beam at its total length, inclusive of all segments. 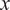 is the total distance from the origin point at varying lengths depending on which MAVS configuration and combination of 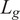 and 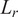 are used to create the final ratios. Each length is broken down and specified individually to account for various ratios of rigid surface versus soft surface area. (b) The theoretical force versus displacement relationship, that is, stiffness, of the MAVS for various values of 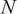 , which denotes the quantity of exposed soft actuators segments in MAVS-A2 design, and the front view of the MAVS-A2 design is illustrated with multiple segments of 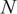 . The applied transverse load is denoted by 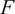 at the free of the MAVS in (a2).

The rigid components of the MAVS actuator were small, thin pieces of custom 3D-printed polylactic acid (PLA), which were embedded into the soft actuator fabric layers during the sewing stages of the fabrication process. The rigid materials limited vertical expansion of the actuator when pressurized and restricted physical internal volume by limiting the cross-sectional area of the MAVS. The rigid retainers were placed along the length of the actuator and alternated with sections of exposed fabric, providing the MAVS with varying levels of compliance. By alternating segments of soft and rigid-bound cross sections, the MAVS obtained varying levels of lateral stiffness that can be adjusted during fabrication and pressurization (Figure 4a).

The rigid retainers were tested in three sizes, labeled 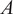 1– 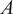 3 for each size of rigid retainer and soft actuator gaps. In this study, only the type 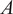 was used. The size of the rigid retainer was 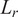 = 1 cm. The gap length was denoted by the numerical value assigned, with *A*1 = 0.5 cm, *A*2 = 1 cm, *A*1 = 1.5 cm. Based on the previous analysis presented in (Thalman et al., 2020), the MAVS-A2 was selected for the SR-AFO. The selected ratio between rigid and soft materials allowed for high stiffness for medial/lateral ankle support when pressurized (inflated) without becoming overly stiff when inactive (deflated).

The MAVS actuator was modeled as a cantilever beam, fixed and pinned in place across one half of the actuator, with the other half free to move. The cross section of each segment of the MAVS was accounted for in the final equation for deflection of the free end. The total deflection 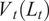 of the MAVS with 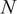 segments of alternating materials was calculated by
(7)

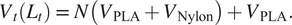



These material types were denoted by the variables 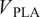 and 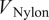 , which indicate the deflections using the material properties of PLA and Nylon, respectively. Segments for PLA ( 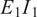 ) and Nylon ( 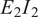 ) respond differently when subjected to external loads, due to differences in material properties. These were accounted for individually for both 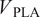 and 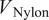 using Young’s elastic modulus ( 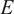 ) and the moment of inertia ( 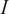 ) of each segment. Each of the rigid (PLA) and soft (Nylon) segments was modeled as a simply supported cantilever beam with a single point load at the free end and using Timoshenko’s theory (Wielgosz and Thomas, 2002; Wielgosz et al., 2008; Thomas and Le Van, 2019). Applying this theory, the deflection of each segment ( 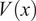 ) can be modeled as
(8)

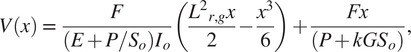

where 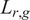 is the length of each segment ( 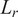 for the rigid segment and 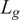 for the soft segment), which can be calculated at a length 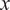 away from the fixed end 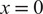 . The beam was subject to an internal pressure 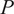 and a transverse force 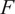 at the free end, where 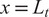 . The second moment of inertia, 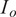 , was determined by the shape of the cross-sectional area and the axis about which the actuator was being deflected. The shear coefficient was represented by 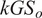 , where 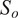 is the cross-sectional area, 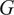 is the shear modulus of the material, and 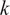 was determined by the cross-sectional shape and Cowper’s formulation (Cowper, 1966).

The maximum deflection was calculated with 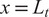 (Wielgosz and Thomas, 2002), which reduced equation (8) to
(9)

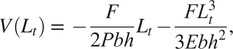

where 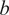 is the base length of the cross-sectional area (which was kept constant at 4 cm), 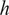 is the height of the actuator when inflated, and 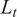 is the total length of the MAVS actuator. The length 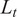 of the actuator was calculated as
(10)

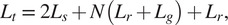

where 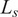 is the length added by the seam, 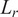 is the length of the rigid piece, and 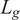 is the length of the gap between rigid pieces where the soft actuator was exposed (Figure 4a1). The number of exposed sections of the soft actuator was represented by 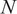 to account for varying lengths of the MAVS. The effects of varying 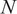 are shown in Figure 4b, where applied transverse load versus tip deflection are shown for varying 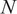 conditions.

### 2.3. Finite Element Analysis

In order to investigate the behavior of each actuator prior to fabrication, simulations were performed using FEA. The analysis predicted the accuracy of the analytical models, validated the behavior of the soft and integrated materials, and optimized the geometric parameters of the design. The FEA simulation was performed using an FEA software (ABAQUS, Dassault Systems, Vlizy-Villacoublay, France) in a dynamic explicit environment. The thermoplastic polyurethane (TPU)-coated Nylon was simulated using Young’s Modulus of 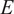 = 498.9 MPa, the Poisson’s ratio of 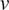 = 0.35, and the material thickness of 0.15 mm. These properties were found in previous studies using the same materials (Thalman et al., 2019), utilizing methods from previous works implementing and characterizing TPU-coated Nylon (Adams et al., 2018).

For each actuator design, the air chambers were modeled by creating a 2D homogeneous thin shell with the net shape of a single layer of the TPU-coated Nylon. Partitions were created where the heat seals were placed. Two layers of the thin shells were stacked in an assembly, and the innermost facing surfaces of each seam partition were bound using a tie constraint. The innermost facing surfaces of the air chambers were designated as a load-bearing face, and a uniform pressure load was applied outward from the initial plane of the fabric in both directions to simulate pressure. This was done for both the ff-PAM and MAVS actuators, which are described in more detail in the following sections.

Initial simulation setup was critical in ensuring that excessive node or element rotations did not occur through the simulation. For simple shapes, basic translational constraints along the fixed edges proved sufficient to keep the thin shell walls from experiencing high levels of nodal rotation or distortion. For more complicated layers such as the MAVS actuators, layers had to be positioned with small gaps between fabric and fixed rigid components to allow proper inflation of each chamber before removing any constraint on rigid parts, to allow for a more natural interaction between the compliant pressurized surface and the solid rigid surface.

#### 2.3.1. FEA analysis of ff-PAM

An FEA model was created for the ff-PAM using two layers of TPU-coated Nylon stacked and tied at the seams. To investigate the force and pressure relationship at a fixed displacement (0 mm), the simulation was performed when actuator vertical displacement at each end was held constant and the pressure was varied. The constant displacement condition was intended to estimate the theoretical maximum force output of the actuators, which occurs when displacement of the actuator was fixed at its original length (Ching-Ping and Hannaford, 1996; Doumit et al., 2009).

**Figure 5.**
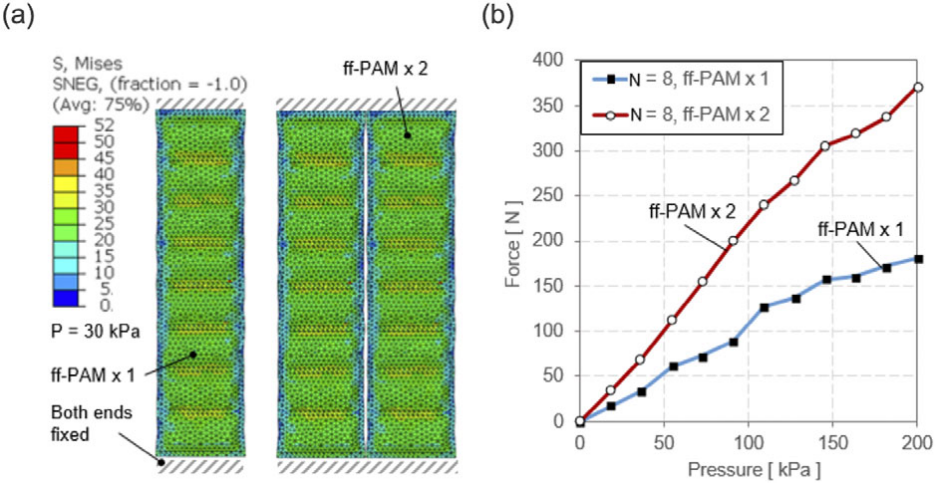
(a) A sample FEA simulation result. Both ends of the actuator are fixed, and the pressure is varied to obtain the maximum tensile force at each level. The color map shows the stress across the surface of the actuator to show a uniform loading of the internal pressure force. A sample result at 30 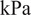 is shown. (b) Simulation results of the force versus pressure relation at the constant displacement for the single and dual ff-PAM actuators. Forces are the result of the summation of vertical force components along the fixed ends.

Pressure was incremented with each simulation until the model stabilized and a final maximum force value was obtained. One end was fixed in all directions, whereas the other was fixed vertically and used to evaluate the reaction forces across its surface to estimate the force. The forces in the vertical direction were summed along the width of the top of the actuator to estimate the tensile force generated from the actuator. This was done for varying pressure levels (0–200 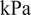 ), as well as for the single and dual ff-PAM actuators (Figure 5a). For the same level of pressure, the dual actuators always exhibited a higher output force than the single actuator (Figure 5b). At the maximum pressure level tested (i.e., 200 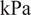 ), the peak force was 180.2 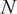 and 373.3 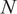 for the single and dual actuators, respectively. Simulation results of maximum force obtainable at each pressure level can be used to estimate the behavior of the actuator prior to fabrication.

#### 2.3.2. FEA analysis of MAVS

The MAVS was modeled using a combination of simulated materials for both the soft actuator and rigid retainers. Thin 2D homogeneous shells were used in the shape of a hollow rectangle to create the pneumatic chamber, with the length and width of the rectangle the same dimensions as that of the MAVS width and length. The rigid retainers were modeled using solid 3D homogeneous extrusions, and assigned a material property for PLA 3D-printed material, which was modeled using material properties with Young’s Modulus of 3,600 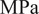 and the Poisson’s ratio of 0.3 as used in previous works (Tehrani et al., 2014; Pepelnjak et al., 2020). More thorough explanation of MAVS FEA modeling can be found in previous studies (Thalman et al., 2020).

The pneumatic pouch was sealed by tying the edges of the thin shells around the perimeter of the rectangular parts. The rigid pieces were placed on the top and bottom faces of the rectangular shell, parallel to the face, and spaced according to which MAVS variation was simulated. An additional 2D homogeneous shell of Nylon fabric was placed to encase the stacked soft actuator and rigid retainers. The outward faces of the rigid pieces were tied to the outer shell, and a global interaction property for surface–surface contact was applied to the assembly. A solid 3D homogeneous clamp was created with the PLA material property and fixed to hold the actuator at a fixed point for the cantilever beam example modeled in the previous section. The major benefit of being able to model the MAVS was to show the interaction between multiple layers of several material types, thicknesses, and properties. This allowed the internal chambers of the MAVS to be observed and studied as done in other works with variable stiffness actuators (Sun et al., 2020).

**Figure 6.**
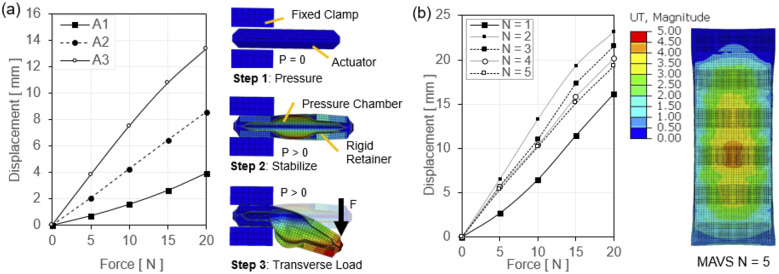
(a) The force–displacement output of a single multi-material actuator for variable stiffness (MAVS) actuator is evaluated with finite element analysis (FEA) for the MAVS-A1, A2, and A3. Various loads are applied to the free end of the MAVS while in a cantilever orientation and the resulting displacement is recorded as the actuator beam begins to buckle under load. (b) FEA simulation results of the same sequence of steps as (a), with the MAVS-A2 actuator, varying the number of segments ( 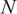 ) for each simulation to calculate the total displacement of the free end.

Two loads were applied to the model: (a) a uniform pressure load to the internal faces of the thin shells of the actuator, and (b) a transverse load applied at a fixed point at the end of the actuator. A total of three steps were run for the simulation: (1) pressurization, (2) stabilization, and (3) point loading as depicted in Figure 6a. The deflection of the MAVS was measured by fixing one half of the MAVS and applying a perpendicular force to the free end. Transverse loads of 5, 10, 15, and 20 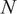 were applied at a fixed point on the free end of the actuator, which was inflated to 100 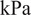 . This was done for the three highest performing MAVS designs ( 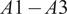 ) predicted by the analytical model. The deflection at the end of the actuator was measured along the direction of the transverse load. Simulation results showed that stiffness of the MAVS decreased as 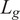 increased (Figure 6a).

Since the MAVS-A2 was selected as the primary MAVS actuator for the SR-AFO, the MAVS-A2 design was evaluated in further detail using FEA to analyze the behavior of the actuator at varying lengths and numbers of segments of alternating materials 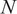 . The MAVS-A2 actuator was modeled in the FEA simulation as a cantilever beam as done in the previous simulation. One end of the MAVS was constrained between two infinitely stiff, fixed blocks which held the end in place. The other end of the MAVS was subjected to a transverse point load at the end of the actuator perpendicular to the top surface as shown in Step 3 of Figure 6a. The tip deflection of the MAVS was recorded for loads of 5, 10, 15, and 20 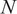 . The value of 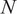 was also increased after each set of point loads was applied from 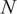 = 1–5 with the internal pressure of the MAVS fixed at a constant 50 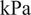 for each simulation (Figure 6b).

Simulation results showed the least deflection with 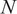 = 1 across all loading conditions, likely due to the small net size of the actuator. However, as 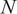 increased more than one, the degree of deflection was comparable for each loading condition. This result helps to validate that, even at longer lengths, the MAVS-A2 actuator can maintain relatively constant bending stiffness and resist deflection against medial and lateral loads. This is a critical point as a single MAVS-A2 chamber is not long enough to cross the length of the human ankle, whereas the MAVS-A2 at 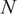 = 5 is able to cross the ankle joint effectively.

### 2.4. Actuator Characterization

A universal testing machine (UTM; Instron 5565, Instron Corporation, High Wycombe, United Kingdom) was used to experimentally characterize both the ff-PAM and MAVS actuators of the SR-AFO exosuit and evaluate their performance.

#### 2.4.1. Characterization of ff-PAM

Three different types of experiments were performed to evaluate (a) tensile force versus contraction at varying pressure levels; (b) tensile force versus pressure at a fixed displacement at 0 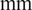 (i.e., zero contraction); and (c) dynamic response of tensile force generation.

**Figure 7.**
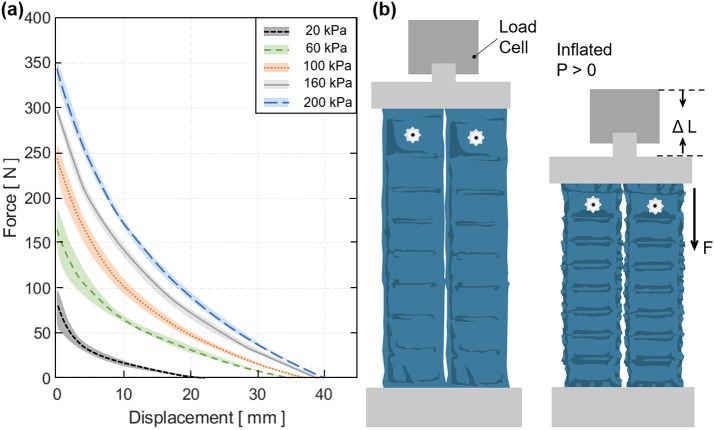
(a) The tensile force output versus contraction of the dual flat fabric pneumatic artificial muscle (ff-PAM) actuator in the quasi-static experiment. Five constant pressure levels are tested, and mean and mean 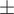 standard deviation are shown. (b) The test conditions and setup of the dual ff-PAM actuator in the UTM before and after pressurization.

In the first quasi-static experiment, the output tensile force versus actuator contraction (or displacement) relation was evaluated under varying pressure levels. The experiment was performed at five different pressure levels (20, 60, 100, 160, and 200 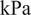 ) with five repetitions per each pressure condition. The UTM was programmed to induce a controlled vertical translation and measure actuator force versus contraction. Each measurement was completed once the load cell reading was 0 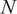 , indicating full actuator contraction. The load cell increased the uniaxial compression at 5 mm/s until it read 0 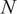 of force. The load cell was returned to the zero position, and the test was run cyclically for five repetitions. The average result (mean and mean 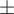 standard deviation [std]) and the test condition are shown in Figure 7a.

Tensile force of the ff-PAM was maximized when the actuator length was maximized (zero contraction), and the force approached 0 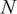 as the actuator fully contracted. In addition, as the pressure level increased, the force output was less variable. At 200 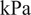 , the maximum force output was 346.5 ± 1.4 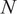 , whereas at 100 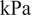 , it was 245.4 ± 15.8 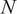 , decreasing the output by 100 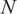 and increasing the variability drastically. Following this trend, at 20 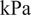 , the maximum force output was 83.0 ± 16.5 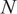 . The variability was close to that at 100 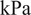 , yet the force dropped drastically.

**Figure 8.**
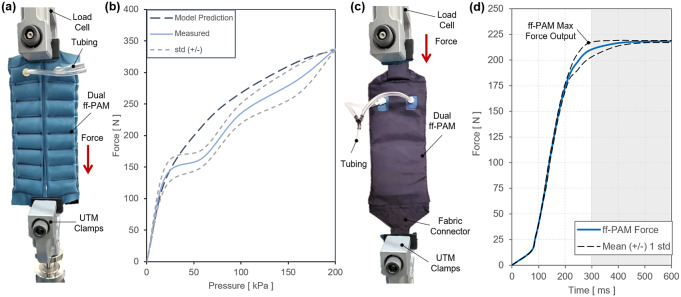
(a) The test setup for experimental characterization of the flat fabric pneumatic artificial muscle (ff-PAM) actuator. The ff-PAM is fixed in the vice clamp of the universal testing machine (UTM). (b) The tensile force output of the dual ff-PAM actuator versus pressure input in the static experiment. Experimental results are compared with the analytic model prediction. (c) The experimental setup of the dual ff-PAM actuator in the casing that attaches the actuators to the soft robotic ankle-foot orthosis. This includes two fabric connectors that affix the ends of the actuators to a single anchoring point. (d) The dynamic response of the dual ff-PAM actuators, while clamped at maximum length in the UTM interface. The response is measured for the time required to achieve maximum force output at 150 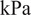 .

In the second static experiment, the output tensile force versus pressure relation was evaluated when displacement was fixed at 0 mm. This relation was compared with the predictions from the analytical model and the FEA simulated maximum force threshold. The dual ff-PAM was placed in a vice clamp with the UTM displacement fixed at 0 mm. The actuators we placed in the UTM are shown in Figure 8a. The pressure increased quasi-statically from 0 to 200 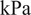 in fixed increments of 10 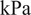 until a stable load could be read from the UTM. Three repeated measurements were performed. The final maximum tensile force output of the ff-PAM actuator at 200 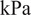 measured by the UTM was 337.1 ± 1.4 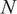 (Figure 8b).

In the third experimental characterization, a dynamic test was performed to obtain a force versus time relation to measure how quickly the actuator reacts to pressurized input. A fabric connector was affixed to the top and bottom of the ff-PAMs to secure both actuators together with a tapered fabric component used to transfer the force from pressurization to a single point (Figure 8c). By allowing the actuator to interface with a fabric anchor, it is assumed that a more realistic force output can be measured to represent the behavior of the actuator when worn on the SR-AFO exosuit. The dynamic response was initiated by rapidly opening a three-way, two-channeled solenoid valve (320-12 VDC, Humphrey, Kalamazoo, MI, USA) to quickly deliver pre-set pressure to the internal air chambers of the ff-PAM. This characterization is important since the ff-PAM actuator is designed to provide a tensile force through uniaxial contraction to the posterior end of the foot to assist plantarflexion during the push-off phase of human gait, and the actuator must be able to provide sufficient force output within a time window that allows the user to feel each controlled perturbation. At a fixed pressure of 150 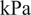 , the valve was opened to provide rapid pressurization. The actuator was able to provide 212.3 ± 7.7 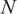 of tensile force in 0.29 s (Figure 8d).

#### 2.4.2. Characterization of MAVS

**Figure 9.**
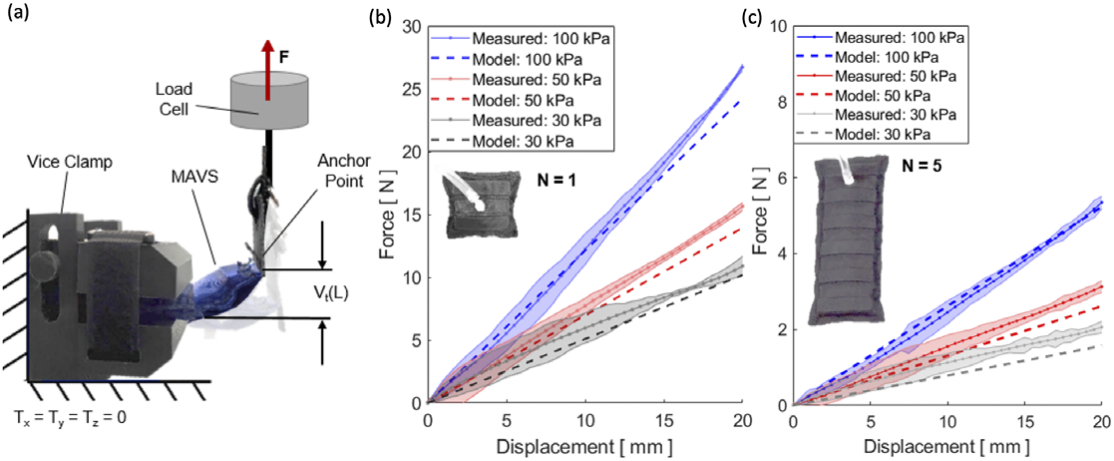
(a) The test setup for experimental characterization of the multi-material actuator for variable stiffness (MAVS) actuator. The universal testing machine is shown from the side view, with the custom 3D-printed clamp and the MAVS actuator fixed to the load cell. (b) Measured force versus displacement/deflection relationship for a single unit of the MAVS-A2 actuator ( 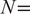 1). A maximum 20-mm deflection was tested at three pressure input levels (30, 50, and 100 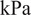 ). Model predictions of the force required to deflect the same distance (20 mm) is shown with dotted lines. (c) Measured force versus the displacement/deflection relationship for a longer MAVS-A2 actuator ( 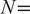 5) used in the soft robotic ankle-foot orthosis.

The MAVS actuators were tested using a custom clamp which was fabricated to fix the actuators in place while being subjected to deflection testing. The MAVS had a tab sewn into the free end to interface with the clamps paired with the load cell of UTM (Figure 9a). This allowed for the UTM to apply a point load to the MAVS, while it was fixed in a cantilever position. The UTM pulled the free end of the MAVS upward 20 mm. The tab acted as a constant point of contact so that the lever arm distance did not change. Each iteration was deflected upward, and the force was measured so that the stiffness of each MAVS could be determined.

The MAVS-A2 actuator, our selection for the SR-AFO, was evaluated for pressure levels of 30, 50, and 100 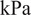 in the cantilever orientation and compared to the analytical model. The rationale for these pressure level selections was based on two factors: (a) accuracy of higher pressures and (b) comfort of the user. With the MAVS actuator integrated into the SR-AFO design, user feedback indicated pressure levels of above 100 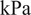 were not comfortable during walking. This evaluation was performed for the original MAVS-A2 design where 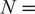 1, and performed a second time for MAVS-A2 for 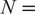 5, the latter of which is the version of the MAVS-A2 actuator embedded into the SR-AFO exosuit.

The MAVS-A2 actuator with 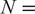 1 experienced 20 mm of deflection with an applied load of 12.1 ± 0.2 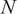 , 15.6 ± 0.1 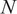 , and 26.7 ± 0.1 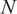 at 30, 50, and 100 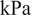 , respectively (Figure 9b). It was observed that as pressure decreased, so did the applied load required to reach the fixed displacement threshold. The variability increased with lower pressures, although this was anticipated as lower pressure would result in higher changes of buckling at unpredictable locations. Increasing the length to 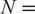 5 showed similar trends. While the overall deflection had less resistance to bending observed than the 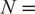 1 condition, this was an expected result since the MAVS-A2 had an increased length. The MAVS-A2 with 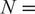 5 required 2.4 ± 0.2 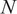 , 3.1 ± 0.2 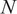 , and 5.3 ± 0.2 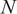 , for 30, 50, and 100 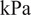 , respectively, to reach 20 mm of deflection (Figure 9c).

### 2.5. Actuator Fabrication

The pneumatic chambers of the ff-PAM and MAVS actuators were fabricated using TPU-coated Nylon fabric (200 Denier Rockywoods Fabrics), which was thermally bonded with a 2-mm heat impulse sealer (AIE-500 2-mm Impulse Sealer, American International Electric Inc., City of Industry, CA, USA). The heat impulse sealer applied uniform heat and pressure to the seam to create an air-tight seal (Thalman et al., 2019).

#### 2.5.1. Fabrication of ff-PAM

**Figure 10.**
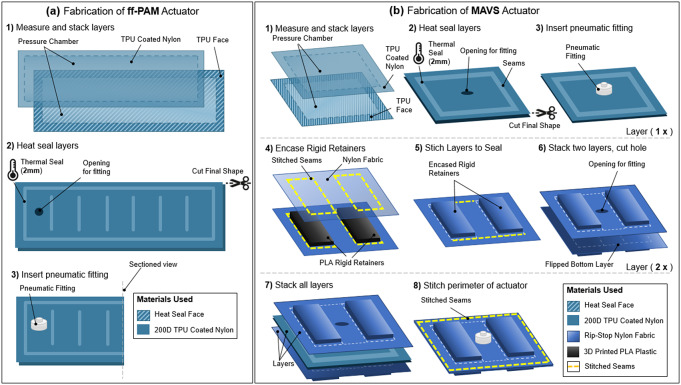
(a) The fabrication process of the flat fabric pneumatic artificial muscle actuator. The formation of the air-tight chamber using thermoplastic polyurethane (TPU)-coated Nylon and a heat seal (a1), the creation of the ribs and placement of the pneumatic fitting (a2), and the final fitting placement (a3) are illustrated. (b) The fabrication process of the MAVS actuator. Heat sealing and fitting placement of the soft actuator using TPU-coated Nylon to form the air-tight chamber (b1–b3), the laying and placement of the rigid retainers in the out layers of the MAVS by embedding polylactic acid (PLA) into Nylon fabric layers (b4–b6), and the final stages of integrating and stacking the MAVS layers (b7 and b8) are shown where the air-tight chamber is placed in between two layers of PLA embedded in Nylon. All components are stitched together around the perimeter to form the MAVS.

The ff-PAM actuator was fabricated primarily following the procedures for soft fabric actuators listed above; however, there were a few additional details that made the actuator design unique. Two layers of TPU-coated Nylon were first stacked and sealed (Figure 10a1). Once three sides were sealed, thick card-stock stencils were placed over the heat sealer with a gap size of the inner seams (Figure 10a2). This ensured that the seal was only formed for the segment in the center and left the gaps open and unsealed on either side of the ff-PAM to allow airflow to the subsequent segments. Each segment was sealed using this technique until the eight chambers were created. A hole was created in the last chamber (Figure 10a2), and a fitting was inserted (Figure 10a3). The last side was then sealed to create one ff-PAM actuator.

For the dual ff-PAM, two actuators were fabricated using this process and laid out in parallel. Nylon fabric (uncoated) was cut to sew the two actuators to one another along the length of the top and bottom seams. A standard tabletop sewing machine (SE-400 Brother, Bridgewater, NJ, USA) was used to create these seams and stitch the fabric connector onto the ff-PAMs. The Nylon fabric connector was cut into a pre-defined pattern that matched the width of the dual ff-PAM and tapered to a single thin strap. This allowed for the dual ff-PAM to be affixed firmly to the SR-AFO at the base of the heel and at the back of the knee with Velcro.

#### 2.5.2. Fabrication of MAVS

The first of the three layer design was composed of rigid PLA 3D Printer Filament (1.75-mm diameter PLA 3D Printer Filament, HATCHBOX, Pomona, CA, USA) sewn between two layers of fabric. Two layers of the embedded rigid retainers were used to encase an inflatable actuator in between. A sewing machine was used to create stitching to hold the rigid retainers in place, as well as to hold the layers together.

The MAVS consisted of a total of three main layers as shown in Figure 10b: a single fabric-based inflatable actuator and two layers of Nylon material with the rigid retainers embedded into the layers. The inflatable chamber was sealed at the designated location to create a rectangular shape using the heat impulse sealer on three of the four sides (Figure 10b1). The fourth side was left open for the installation of the pneumatic fitting (Figure 10b2). A small hole was cut into the fabric, and the threaded Nylon barbed nozzle and nut fitting were secured onto the TPU-coated Nylon (Figure 10b3). The final side was sealed with the impulse sealer to create an air-tight seal that is the same net shape as the entire actuator.

The additional two layers were fabricated using the same method for each (Figure 10b4–b6). The rigid retainers were 3D-printed using PLA and have a thickness of 2 mm and a width of 40 mm, whereas the length as well as the distance between the rigid retainers were 10 mm for the selected MAVS-A2 actuator. Each of the constraining layers was made from two pieces of Nylon fabric, which were stacked with the rigid retainers placed in between at fixed distances. A sewing machine was used to create a stitched seam around the net shape of the rigid retainers, encasing the parts between the two Nylon layers. This was done to create the top and bottom constraining layers. A hole was cut into the top constraining layer to allow the tube fitting from the soft actuator to fit in between the rigid retainers. The sealed soft actuator was placed in between the two constraining layers (Figure 10b7), with the fitting centered within the hole cut previously into the top constraining layer. A final seam was sewn in a rectangular shape around the rigid retainers, at a 5-mm offset. This seam allowance provided a buffer to avoid sewing into the sealed soft actuator and to provide an offset that constrained vertical expansion during inflation (Figure 10b8).

### 2.6. SR-AFO Hardware Design

**Figure 11.**
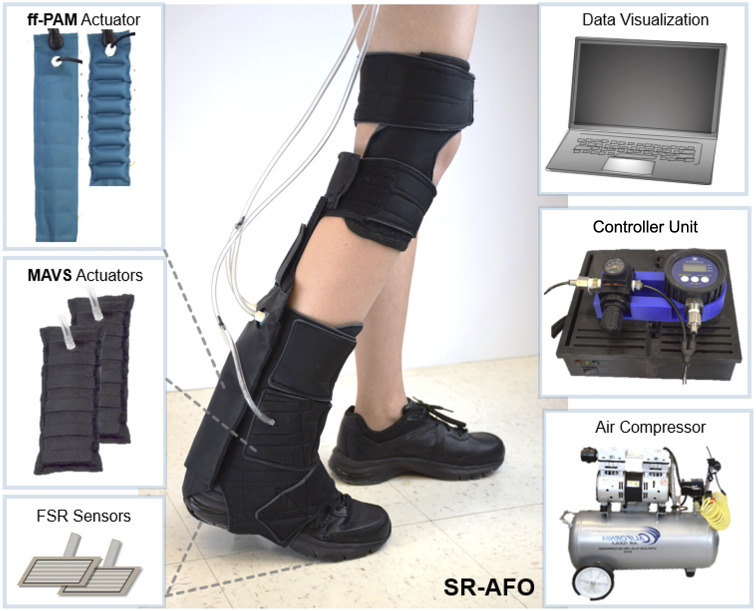
(Middle) The final assembly of the soft robotic ankle-foot orthosis and its components. (Left) The flat fabric pneumatic artificial muscle and multi-material actuator for variable stiffness actuators and the force-sensitive resistor sensors for gait detection. (Right) The controller, pneumatic source (air compressor), and visual feedback for system operation.

The SR-AFO was designed to be worn in a variety of applications, and is shown with all critical components in Figure 11. The hardware used to control the exosuit is housed within a lightweight fabric belt, which can be adjusted to be worn on the hips, or worn as a backpack depending on the user preference. The cables are long enough to allow the hardware to be set aside and placed next to the test platform if on-board hardware is not ideal for the test conditions. The tabletop version of the design was used in this study. The total worn mass of the SR-AFO system is 0.203 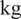 . The compressor is not worn during use. The compressor and all hardware sit beside the treadmill and/or walking path with a tether to the participant.

The hardware logic controller used an Arduino Mega 2560 Rev3, which connected all analog inputs and digital outputs of the SR-AFO to monitor the status of the system. These I/O were categorized by the force-sensitive resistor sensors (Interlink 406, Adafruit, New York, NY, USA), which were embedded in the user’s shoe to detect gait phase. Pressure sensors (ASDXAVX 100PGAA5, Honeywell Sensing and Productivity Solutions, Charlotte, NC, USA) were used to monitor actuator pressure throughout the operation of the SR-AFO, and three-way, two-channeled solenoid valves (320-12 VDC, Humphrey) MOSFETs (IRF520 MOSFET Driver Module) were used to actuate the SR-AFO exosuit at various times. The control pouch was connected to a portable air compressor (Model 8010A, California Air Tools, San Diego, CA, USA), which can be easily placed next to the current test site or facility and provided a pneumatic power source for the actuators.

## 3. Human Trials and Experimental Evaluation of the SR-AFO

In order to evaluate the effectiveness of the SR-AFO, specifically the MAVS for lateral ankle support and the ff-PAM for plantarflexion assistance, three different human experiments were designed and performed: (a) quiet standing; (b) walking over compliant surfaces; and (c) treadmill walking. A total of six able-bodied participants ( 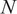 = 6) participated in the experiments (male = 4, female = 2, age = 23–29 years, height = 1.68–1.88 m, weight = 47.6–72.9 kg, BMI = 16.9–23.0, and leg length = 0.81–1.05 m). All the participants gave informed consent prior to participation, and the study was approved by the ASU Institutional Review Board (STUDY00012099). A screening process was used to ensure this study included only able-bodied individuals, with no diagnosed musculoskeletal disorders, gait impairments, or past or current injuries to the lower limbs. Participants with any active illness or symptoms were also excluded.

### 3.1. Lateral Ankle Support during Standing: MAVS

#### 3.1.1. Experimental setup and protocol

The objective of this experiment was to evaluate the effectiveness of the MAVS of the SR-AFO to support lateral ankle stability during standing. The degree of stiffness increase in the frontal plane with MAVS actuation was quantified and compared with natural ankle stiffness in the frontal plane without the exosuit. Sagittal plane stiffness was also quantified to evaluate the potential impact of MAVS actuation on ankle movement in the sagittal plane.

**Figure 12.**
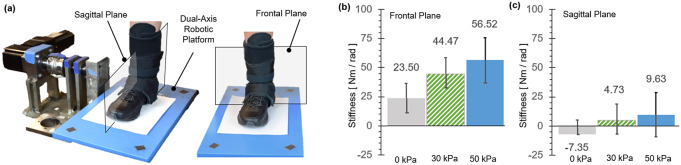
The dual-axis robotic platform setup (Nalam and Lee, 2019) is shown with the user wearing the soft robotic ankle-foot orthosis exosuit for the quantification of ankle stiffness in (a) the sagittal plane and the frontal plane. (b) Average ankle stiffness in the frontal plane (with eversion perturbations) and the sagittal plane (with dorsiflexion perturbations) under different exosuit support conditions. The recorded findings are all in reference to the baseline measurement without the exosuit.

Each subject wore a pair of custom athletic shoes, and a dual-axis goniometer (SG110, Biometrics Ltd., Ynysddu, UK) was placed on the right foot-ankle complex to measure 2D ankle kinematics. The dual-axis robotic platform (Figure 12a), capable of applying position perturbations to the ankle in the sagittal and frontal planes and measuring the corresponding ankle torques, was used to quantify 2D ankle stiffness in both the sagittal and frontal planes. The platform was validated to accurately quantify 2D ankle stiffness during upright standing (Nalam and Lee, 2018; Nalam and Lee, 2019; Adjei et al., 2020; Nalam et al., 2020). The subject was asked to stand with the right foot placed on the robotic platform and the left foot on the elevated ground right next to the platform. The right foot was placed in a fashion to ensure that the axes of rotation of the robotic platform were as closely aligned as possible with those of the ankle. Our previous study confirmed that any potential misalignment in the foot placement has a minimal impact on the quantification of ankle stiffness in the sagittal and frontal planes (Nalam and Lee, 2019).

A fast ramp-and-hold position perturbation of 3° and a duration of 100 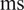 was randomly applied either in the dorsiflexion direction or the eversion direction to quantify sagittal plane stiffness and frontal plane stiffness, respectively. A total of 30 perturbations was applied in each direction. The experiment was performed under four conditions: (a) no exosuit; (b) passive exosuit (at 0 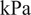 ); (c) active exosuit (30 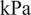 ); and (d) active exosuit (50 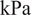 ).

#### 3.1.2. Data analysis

Ankle stiffness was quantified by fitting a linear second-order model, consisting of ankle stiffness, ankle damping, and foot inertia, to the measured ankle kinematics and torques due to perturbation for a window of 100 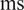 starting from the onset of the perturbation. To check the reliability of stiffness estimation with the second-order model, the percentage variance accounted for (%VAF) between the estimated ankle torque from the best-fit second-order model and the measured ankle torque due to perturbation was calculated (Lee et al., 2014; Nalam et al., 2020). For each subject, stiffness increase with the exosuit was calculated with respect to the baseline measurement without the exosuit, that is, no exosuit condition. Group average results (mean ± std) of the six subjects were reported.

#### 3.1.3. Results

The MAVS of the SR-AFO effectively increased ankle stiffness in the frontal plane with a minimal impact on the stiffness in the sagittal plane (Figure 12). Ankle stiffness was reliably quantified and successfully estimated by the second-order model in all experimental conditions, evidenced by high %VAF, which was greater than 97.5% in any of the eight experimental conditions and in any subjects.

In the frontal plane, simply donning the exosuit (passive exosuit condition) increased ankle stiffness by 23.5 ± 12.6 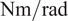 from the free-foot baseline. Activating MAVS of the exosuit significantly increased the ankle stiffness. At the pressure level of 30 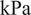 , the increase from the baseline was 44.5 ± 13.8 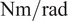 . The stiffness increased with increasing pressure. At 50 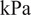 , the increase was 56.5 ± 18.6 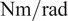 . In the sagittal plane, the change in ankle stiffness was minimal. In average across subjects, even activating MAVS increased the ankle stiffness less than 10 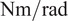 . At the pressure level of 30 and 50 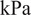 , the stiffness increase from the baseline was only 4.7 ± 14.8 and 9.6 ± 17.4 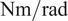 , respectively.

### 3.2. Lateral Ankle Support during Walking: MAVS

#### 3.2.1. Experimental setup and protocol

The objective of this experiment was to evaluate the effectiveness of the MAVS of the SR-AFO to support lateral ankle stability during walking over compliant surfaces. The degree of lateral ankle deflection with MAVS actuation was quantified and compared with the ankle deflection without the exosuit.

The dual-axis robotic platform was used to simulate compliant surfaces in the frontal plane. Our previous study confirmed that the robotic platform was capable of accurately simulating a wide range of compliance (inverse of stiffness) in both the sagittal and frontal planes (Nalam and Lee, 2019). In this experiment, two different compliant surfaces were simulated with stiffness of 100 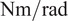 (compliant) and 50 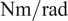 (more compliant) in the frontal plane, whereas a rigid surface (stiffness of 10,000 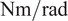 ) was simulated in the sagittal plane.

**Figure 13.**
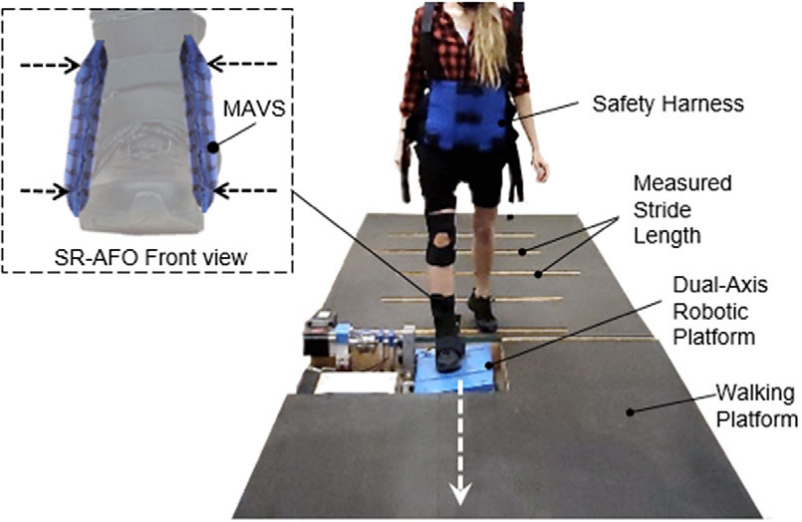
The instrumented walkway setup to investigate the effectiveness of the soft robotic ankle-foot orthosis with multi-material actuator for variable support (MAVS) actuation for lateral ankle support during walking over compliant surfaces. The dual-axis robotic platform utilized two conditions for compliance in the lateral direction for the ankle as the participant walked across the platform, providing randomized levels of surface compliance each time the participant steps on the platform.

The subject was instructed to walk on the elevated walkway (approximately 6 m in length; Figure 13). A metronome was played at 100 bits per minute to encourage a consistent walking cadence. In addition, the subject’s stride length was measured and marked along the walkway leading up to the platform to ensure consistent foot landing on the platform. The experiment was performed under six conditions: 2 surface conditions (compliant and more compliant) × 3 exosuit conditions (no exosuit, passive exosuit (0 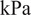 ), and active exosuit (30 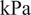 )). In each of the six experimental conditions, 30 walking trials were completed, resulting in a total of 180 walking trials. The order of the surface conditions was fully randomized.

#### 3.2.2. Data analysis

Lateral ankle deflection in the frontal plane was measured using the goniometer from the moment of heel strike to toe-off (0–60% of the gait cycle). To remove outlier data due to simple human error in foot placement on the platform during walking, only the data with the foot center of pressure within 0 (the axis of rotation of the platform) and 5 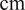 lateral offset were included in data analysis. For each subject, peak-to-peak ankle deflection in the frontal plane was quantified throughout the stance phase, and group average results of the six subjects for this measure were compared across the different support conditions.

#### 3.2.3. Results

**Figure 14.**
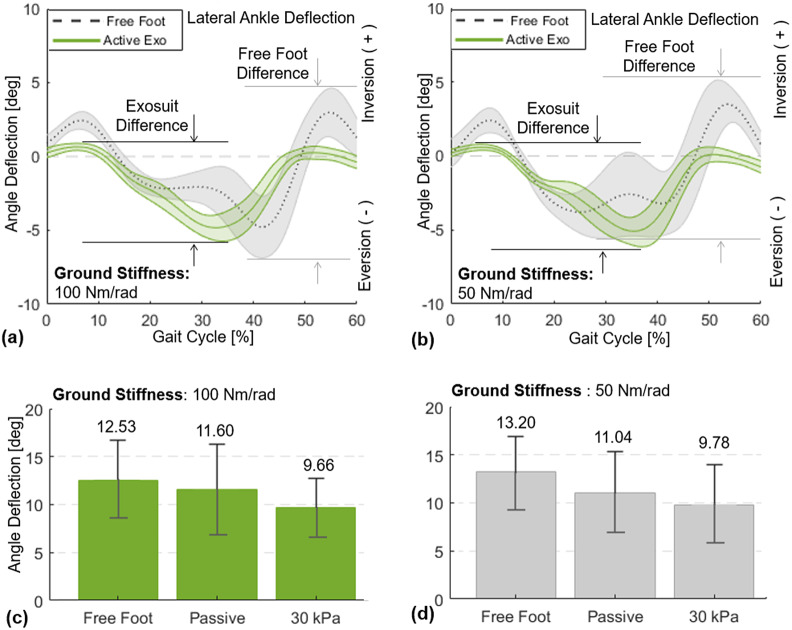
Lateral ankle deflection in the frontal plane for 0–60% of the gait cycle, from the moment of heel-strike to toe-off. Results of a representative subject (with the median change in ankle deflection) are presented for free foot and active support (30 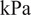 ) when the platform stiffness is set to (a) 100 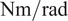 and (b) 50 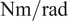 . Group average results ( 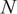 = 6) of the peak-to-peak lateral ankle deflection are presented for free foot, passive (0 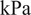 ), and active support (30 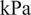 ) when the platform stiffness is set to (c) 100 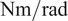 and (d) 50 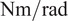

The SR-AFO with MAVS actuation effectively supported lateral ankle stability during walking over the compliant surfaces (Figure 14). Results from a representative subject confirmed a notable reduction in the peak-to-peak ankle deflection in the frontal plane (Figure 14a,b). Group results further demonstrated that these trends were consistent across subjects (Figure 14c,d). In the compliant surface condition (100 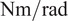 ), the peak-to-peak ankle deflection was 12.5 ± 4.1° with free foot, 11.6 ± 4.7° after donning the SR-AFO (passive exosuit), which was only a minor decrease. However, MAVS actuation with 30 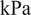 decreased the deflection to 9.7 ± 3.1°. In the more compliant surface condition (50 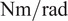 ), the free-foot peak-to-peak ankle deflection was 13.2 ± 3.7°. After donning the exosuit, the deflection was 11.0 ± 4.3°. With MAVS actuation at 30 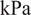 , the deflection decreased to 9.8 ± 4.2°.

### 3.3. Plantarflexion Assistance during Walking: ff-PAM

#### 3.3.1. Experimental setup and protocol

The objective of this experiment was to evaluate the effectiveness of the ff-PAM of the SR-AFO to assist plantarflexion during walking. Activation of plantarflexor muscles in the push-off phase with and without ff-PAM actuation was compared.

**Figure 15.**
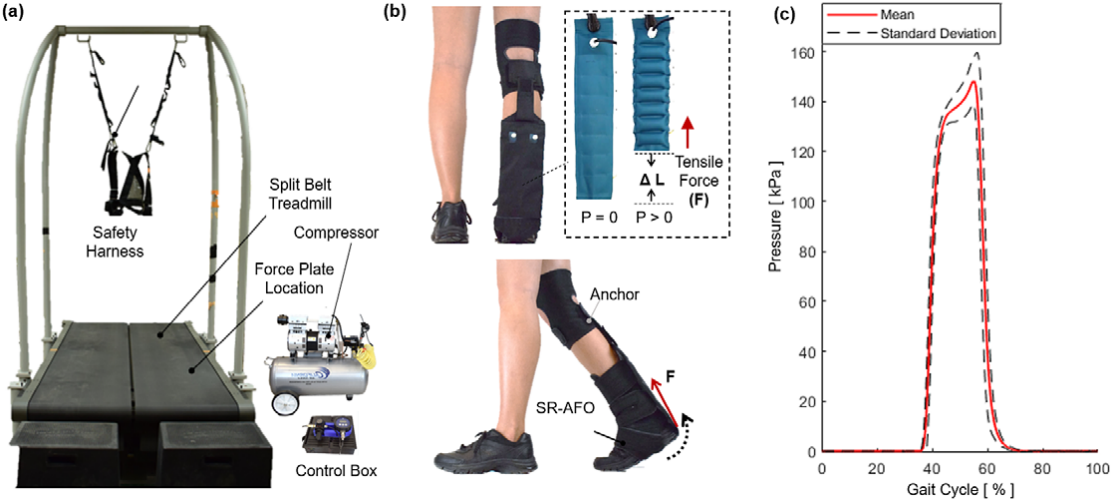
The experimental setup to investigate the effectiveness of the soft robotic ankle-foot orthosis with flat fabric pneumatic artificial muscle (ff-PAM) actuation for ankle plantarflexion assistance during walking. (a) The split belt treadmill with the safety harness, as well as the compressor and control box, which sit beside the testing area. (b) The ff-PAM placement is shown where the dual actuator setup is paired with the fabric connector to run between the back of the knee and the base of the heel. Tensile force applied to the posterior end of the foot to generate a torque about the ankle. (c) Pressure levels are monitored throughout the walking experiment, and measured actuation pressures as a function of gait phase during walking are recorded.

An instrumented treadmill (Bertec Treadmill, Columbus, OH, USA), capable of measuring ground reaction forces (Figure 15a), was used to detect the moment of heel strike and determine the actuation timing of the ff-PAM, where the valve releases instantaneous pressure from 40 to 60% of the gait cycle (Figure 15c). A wireless electromyography (EMG) system (Trigno, Delsys, Natic, MA, USA) was used to monitor activation of two major plantarflexors, soleus (SOL) and medial gastrocnemius (GAS), throughout the experiment. The surface EMG sensors were placed, and maximum voluntary contraction (MVC) of each muscle was measured as per standard International Society of Electrophysiology and Kinesiology protocols (Merletti and Di Torino, 1999).

Prior to the main walking experiment, the subject was asked to select a preferred walking speed, which was determined by increasing the treadmill speed by 0.1 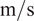 until the subject indicated the pace was too fast for a natural cadence, and then decreased the speed by 0.1 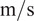 until the pace was determined to feel too slow. This process was repeated one more time, and the final preferred walking speed was selected by averaging the two values. For the subjects in this study, this speed ranged from 0.9 to 1.2 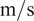 . The subject was then instructed to walk with the selected preferred walking speed for 2 min, which determined the average stride time (i.e., gait cycle duration; 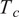 ).

The main experiment was performed under two conditions: (a) no exosuit and (b) active exosuit. In the active exosuit condition, the ff-PAM was pressurized at 150 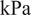 in 40–60% of the gait cycle (0.4–0.6 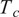 ) and depressurized in the rest phases of the gait cycle, which was designed to assist push-off in the late stance phase. The subject walked for 5 min for each experimental condition. A minimum of 3-min resting period was provided between trials to prevent any potential muscle fatigue.

#### 3.3.2. Data analysis

Muscle effort was quantified by calculating the normalized EMG amplitude. Surface EMG data were first demeaned, rectified, and filtered using a low-pass second-order Butterworth filter with a cutoff frequency of 5 Hz. Muscle activity was then normalized with respect to the maximum muscle activity captured during MVC measurement. The amplitude data were segmented based on successive heel strikes, and each segmented stride data were normalized to the percentage gait cycle (0–100%).

**Figure 16.**
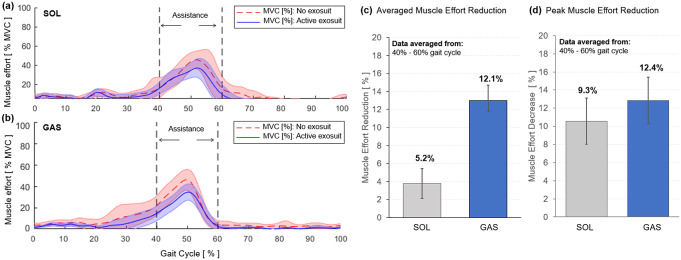
Muscle effort, quantified by the normalized EMG amplitude, of plantarflexors during walking with (solid blue) and without exosuit (dotted red) assistance. Results for the (a) SOL and (b) GAS of a representative subject are shown. The region of applied assistance is indicated at 40–60% of the gait cycle. Group average results of (c) average muscle effort reduction and (d) peak muscle effort reduction are shown for SOL and GAS.

The active region for exosuit assistance (40–60%) was then isolated to evaluate the effectiveness of the ff-PAM for providing assistance to the primary plantarflexor muscles (i.e., SOL and GAS) during push-off (Figure 16). The average reduction of muscle activation in SOL and GAS was quantified by taking the integral of the area under the amplitude curve between 40 and 60% of each gait cycle to determine the difference between the no exosuit and active exosuit conditions. In addition, the reduction in peak EMG amplitude within the assistance time window was calculated between the two experimental conditions. Group average results of the six subjects for these two measures were reported.

#### 3.3.3. Results

The SR-AFO with ff-PAM actuation effectively reduced muscle effort in plantarflexors in the push-off phase of walking. Results from a representative subject showed a reduction in both the average and peak EMG amplitude within the assistance time window (40–60% of the gait cycle) in both SOL and GAS muscles (Figure 16a,b). Group results demonstrated that this trend was consistent across all subjects. Compared to the no exosuit condition, the active exosuit condition reduced the average EMG amplitude by 5.2 and 12.1% in SOL and GAS, respectively (Figure 16c). The peak EMG amplitude was also reduced by 9.3 and 12.4% in SOL and GAS, respectively (Figure 16d).

## 4. Discussion

This paper presents the fully integrated version of the SR-AFO, which is designed to provide medial and lateral ankle support to prevent ankle sprains, as well as plantarflexion assistance to aid the push-off phase during walking. The SR-AFO is made of lightweight materials, entirely fabricated from textile fabrics. The total weight for fabric boot, knee brace, fabric actuators, and tubings is 0.23 kg. The final design considerations present a novel approach to ankle assistance in both the sagittal and frontal planes using two sets of actuators. The focus of this work is for rehabilitative settings and applications, where pressure lines are assumed to already be established and functional within the walls of the facility, and ready for use. The weight of the compressor and control system do not contribute to the weight of the orthosis because this iteration of the design is not intended to have the user don those components of the system. The intention is to have the control box and compressor sitting beside the treadmill or walkway, with a tether connecting the heavier hardware to the participant. All trials reported in this paper were performed in this orientation.

The first actuator presented was the ff-PAM, which contracts similarly to a muscle when activated. The pneumatic actuation, used to create the tensile force generated by the ff-PAM, generates a high force-to-weight ratio that showed promising results for assisting ankle plantarflexion. The number of chambers used in the ff-PAM was set to 8, considering overall length, contraction ratio, and robustness of the actuator. Adding more chambers to each actuator improved the stroke length of the actuators. Previous works showed that the chamber sizing and ratio has the larger impact on the overall force output and contraction ratio, and the number of chambers did not make a notable impact on overall force production with similar design (Niiyama et al., 2015; Thalman et al., 2019; Kwon et al., 2020). Adding a second actuator in parallel is what increased the force significantly. The dual ff-PAM configuration was used to enhance plantarflexion assistance, generating a maximum force output of 337.1 ± 1.4 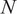 at 200 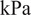 , which fell within 0.5% of the predicted values from the analytical model (336 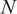 ) and FEA simulation result (337.5 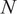 ). Similar designs in previous work (Niiyama et al., 2015) reported a tensile force of 100 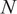 at 40 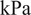 with a single actuator, while the dual ff-PAM achieved 200 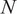 at 50 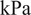 with two parallel actuators. The ff-PAM actuators, made of a TPU-coated Nylon, have a much higher burst pressure than TPU alone (can be pressurized up to 300 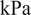 before starting to experience seam failures; Thalman et al., 2019).

In previous work (Thalman et al., 2020), the dual ff-PAM actuator used in the SR-AFO exosuit could provide peak force output of 118.2 ± 3.1 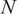 in 0.3 s at 150 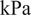 . In this study, after increasing the tubing diameter used in the valves to 1/4 inch outside diameter tubing, the dual ff-PAM actuator was able to provide 212.3 ± 7.7 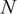 of tensile force in 0.3 s at 150 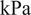 . This yielded a 79.5% increase in the force output from the previously tested design of the SR-AFO. Assuming these force output values, and the average lever arm of 10 cm from the base of the heel to the center of the ankle joint, the estimated torque values correspond to roughly 21.2 Nm at the ankle joint to assist plantarflexion. Previous studies using a rigid ankle robot reported 23 Nm of maximum torque with a weight of 3.6 kg (Roy et al., 2009), which highlights the substantially higher torque density of the SR-AFO.

The MAVS actuator is the second set of actuators featured in the SR-AFO. This design consists of a combination of soft and rigid materials to achieve a design that is compliant when inactive, but retains an increased level of stiffness and resistance to buckling when inflated, fortified by the rigid retainers which limit outward expansion of the soft materials. The design for the MAVS actuators were inspired by previous work done in laminate-based actuators with variable stiffness (Jiang and Gravish, 2018), and were modeled based on Timoshenko’s theory (Wielgosz and Thomas, 2002; Wielgosz et al., 2008; Thomas and Le Van, 2019). This interaction between rigid and soft materials results in the MAVS actuator, which was optimized and characterized for the SR-AFO exosuit. The ratio of rigid to soft material in the final implementation was selected based on the pairing that resulted in low stiffness for user comfort when deflated, and high stiffness for sufficient medial/lateral ankle support when pressurized. The final MAVS design determined to be the best suited for the SR-AFO was the MAVS-A2, with a rigid retainer of 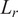 = 1 cm and a gap of exposed soft actuator at 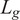 = 1 cm. MAVS-A2 was the second highest stiffness value observed, reaching 26.7 ± 0.1 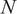 with a calculated stiffness of 1,335.5 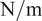 and fell within 9.1% of the model predictions, and also showed the lowest stiffness in the passive condition as presented in previous evaluations (Thalman et al., 2020).

Six able-bodied young participants were recruited to perform a series of experiments to validate the overall efficacy of the SR-AFO. The first performance evaluation was conducted using a dual-axis robotic platform, which characterized ankle stiffness as done in previous works with the participant staying in a quiet standing position (Adjei et al., 2020; Nalam et al., 2020). The MAVS actuators were evaluated using the platform with the SR-AFO donned on the right foot in three conditions: passive (no pressure) and two pressure levels. The group results indicated that the MAVS actuators showed an increase in ankle stiffness in the frontal plane when the MAVS actuators were active, whereas stiffness in the sagittal plane remained fairly constant. The frontal plane showed an average increase of 56.5 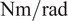 at 50 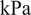 from the baseline free-foot stiffness and 44.5 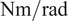 increase at 30 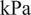 . The sagittal plane showed an increase in stiffness less than 10 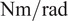 for each pressure level. These results support the proposition that the MAVS actuators can increase ankle stiffness in the medial and lateral directions in the frontal plane, with minimal impact on the range of motion or movement of the ankle in the sagittal plane.

The MAVS actuators were evaluated in a more dynamic setting, where the participants were instructed to walk across the platform, while compliant surfaces were simulated in the frontal plane. Two compliant surfaces were evaluated, at 50 and 100 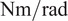 . This was done for 30- and 50- 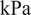 conditions for the MAVS actuators, as well as the passive (0- 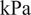 ) condition. Results from these trials showed that the overall peak-to-peak ankle deflection in the frontal plane decreased when the MAVS actuators were active compared to the free-foot condition. The decrease in ankle deflection supports the hypothesis that the MAVS actuators would be able to help prevent ankle sprains. By decreasing the overall deflection of the ankle during walking over an unanticipated compliant surface under the foot, the MAVS demonstrated effectiveness in decreasing the range of motion in the medial and lateral directions. With MAVS actuation at 30 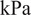 , the deflection decreased from 13.2 to 9.8° in the most compliant condition.

Previous work has proved that restraining inversion–eversion ankle motion can effectively prevent ankle sprains. When compared with no prevention, passive support from ankle taping and bracing significantly reduces the incidence of ankle sprains (Verhagen and Bay, 2010), which has been mainly attributed to the increased ankle stiffness with the addition of external support (Fayson et al., 2013). Considering the small range of motion in inversion–eversion during normal walking (<15°), reduction of about 2.5° is a meaningful decrease. The MAVS actuator was only at a single pressure level of 30 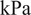 in this preliminary study, and still the SR-AFO provides much higher stiffness support with higher pressure (Figure 12b,c) that would lead to more decreased ankle deflection. The SR-AFO with MAVS actuator has this capability, which is not achievable in traditional passive AFOs.

The dual ff-PAM actuators were evaluated during treadmill walking. EMG sensors were used to monitor muscle activation of the SOL and GAS throughout the trials. The results showed that the SR-AFO was able to reduce the muscle activation of the both plantarflexor muscles during the 40–60% window of the gait cycle in terms of average muscle effort and peak muscle effort. These observed changes and reductions in muscle effort suggest that the dual ff-PAM actuator was able to offset some of the effort required from the participant in the free-foot condition and provide assistance from the robotic actuation during the identified range of the gait cycle, that is, the push-off phase of walking. This result obtained from able-bodied participants is a promising result that could indicate potential benefits when used with impaired users. With able-bodied users, the SR-AFO was able to offset existing effort exerted; however, in impaired users, it is predicted that the SR-AFO would instead be able to supplement an otherwise deficient effort that could be exerted from an impaired limb. Since the average gait cycles measured for able-bodied participants in the study ranged from 1.1 to 1.2 s, the actuation time that reached peak force output within a 0.3-s window would be sufficient to provide plantarflexion assistance with minimal latency.

The most frequently observed modes of failure originated from the heat seals on the actuators. During fabrication, the MAVS actuators had the potential to fail due to human error in the precision needed in sewing the actuators together in order to create a tight and compact form factor in the design. However, once sealed and sewn together correctly, no failure of the MAVS actuators was observed during the trials. The ff-PAM actuators showed signs of fatigue in the heat seals, mainly the center seals that separate each segment. The seams would periodically burst after several days of rigorous use and testing with participants, where the heat seal would delaminate, rupture, and prevent proper inflation. The ff-PAM actuators are simple to fabricate and recreate (approximately 10 min) and with the design of the attachment points on the SR-AFO, only take a few minutes to swap out with a new actuator if a burst did occur.

The SR-AFO showed promising results when evaluated with able-bodied participants in both standing and walking conditions. Overall evaluation of the SR-AFO exosuit showed a potential for future trials with impaired users to assist in lower extremity tasks and serve as a preventative measure to reduce risk of trips or falls due to lateral ankle instability.

Given the potential of this study, several limitations should be acknowledged. The main focus of this paper was design, modeling, and fabrication, and preliminary testing of the device. This study reports the results of six subjects, which is too low to perform and report accurate or reliable statistical analysis. Rigorous human experiments to evaluate the effectiveness of the device will be performed in our future studies. Preliminary results of the design were promising, but further and more expansive studies need to be conducted to report reliable statistical significance of results. Other limitations included in-lab testing rather than the ability to test in a clinical setting. Even with an adjustment round to acclimate to the device, we anticipated slight changes to the reported kinematics in this study. We plan to investigate this over a wider range of able-bodied participants in future work and monitor kinematics over a larger sample size. Additionally, this study evaluated only eversion extensively during static standing, and so future work will investigate effectiveness of the MAVS actuators to increase inversion stiffness at the ankle.

Future work for the SR-AFO will begin to investigate the benefits of the device in clinical trials with users suffering from various gait abnormalities. Clinical trials will be a critical next step to begin identifying the rehabilitative capabilities of the device. Other future efforts will focus on reducing the size and weight of the control box to provide a more comfortable and low-profile design for increased comfort and portability. Additional future work will also investigate the metabolic cost of walking while using the SR-AFO to determine the total reduction in effort to expand on EMG data collection during use. Finally, ongoing and planned research efforts have begun utilizing the SR-AFO for entrainment studies (Thalman et al., 2021), and expanding the actuation and assistance of the actuators to other joints such as the hip (Thalman et al., 2021; Baye-Wallace et al., 2022) and comparing results to other rigid exoskeleton robots.

## Data Availability

Data availability is not applicable to this article as no new data were created or analyzed in this study.
